# Supplementary material for: Are carbon emissions trading and green financial instruments synergistic? -Comprehensive quantitative research based on content analysis
Source: PLoS One. 2024 Mar 7;19(3):e0298601. doi: 10.1371/journal.pone.0298601 (PMC10919601; doi:10.1371/journal.pone.0298601)
Supplement: S1 Data — (ZIP) [file pone.0298601.s001.zip › Data/地级市Z_F工作报告.zip!/║■─╧/┬a╡╫╩╨/└┤╫╘╣1⁄2╓┌║┼í╛┬φ┐╦╩2╛▌═°í┐_2018.docx]

各位代表：

现在，我代表市人民政府，向大会作政府工作报告，请予审查，并请各位政协委员和列席人员提出意见。

一、2017年工作回顾

刚刚过去的2017年，是充满挑战但喜获丰收的一年。在市委的坚强领导下，全市上下认真贯彻落实党中央、国务院和省委、省政府的决策部署，紧紧围绕建设富饶美丽健康祥和新娄底的目标，大力实施创新引领开放崛起战略，坚持加速转型、奋力赶超，全面推进七个“三年行动计划”，坚决打好“三大攻坚战”，努力开创了全市经济社会发展的新局面。

经济发展稳中趋好，越来越好。1-11月，纳入省政府绩效考核的13项主要经济指标，我市第一、二产业、规模以上工业、建筑业等4项指标，增速排全省第一位；固定资产投资增速排全省第二位；地区生产总值增速排全省第三位；其中11项指标，增速高于全省平均水平。预计，全年实现地区生产总值1560亿元（预计数，下同），增长8.5%左右，一、二、三产业分别增长3.5%、8%、11%左右；固定资产投资增长14.7%左右；规模工业增加值增长8.6%左右；社会消费品零售总额增长10.8%左右；一般公共预算收入达到115.63亿元，增长10.55%；城乡居民人均可支配收入分别增长8.5%、11%左右；规模以上工业企业新增113家，净增59家，利润增长73.7%；税收占财政收入比重达到59.41%。在全省竞相发展、你争我赶的发展大势中，彰显了娄底力量。

一年来，我们坚持工作高标准，全力保重点、攻难点、创亮点。一是在推进脱贫攻坚、生态环境治理、安全生产等重点工作中，坚持不怕困难、不怕麻烦、不怕出丑的“三不”精神，发动群众广泛参与，敞开大门抓整改，打赢了年度攻坚战。二是面对历史罕见的洪涝灾害，全民动员，科学应对，众志成城，紧急转移群众16.37万人，解救受困群众4740人，夺取了防汛避险救灾的全面胜利。三是强力推进中心城区道路提质改造、路网完善及风貌整治，吉星路、娄星南路、甘桂路等9条“断头路”建成通车，湘阳街、乐坪西街、扶青路等10条道路完成“白改黑”，顺利通过国家卫生城市复查评审，以全省第一名的成绩保留了全国文明城市提名资格。四是行政区划调整平稳有序推进，涟源市水洞底镇、双峰县蛇形山镇划入娄星区管辖，新化县撤销上梅镇和上渡办事处，改设3个街道。五是在全国率先推进健康快车下基层，在全省率先完成农村墙体广告清零、率先实现市县乡村四级公共法律服务全覆盖。六是成功跻身全国产业转型升级示范区，全面启动国家级高新区创建，全省产业发展现场推进会首站观摩放在娄底，我市作典型发言。这些做法和成效，集中展示了我市发展的新格局和新形象，极大提升了娄底的对外影响力和美誉度。

一年来，我们以“跳起来摘桃子”的勇气，按照“四个略高于”的要求，较好地完成了年初确定的各项目标任务。

（一）项目建设推进有力。继续开展“项目建设大比拼”活动，推进各类项目2800个，完成固定资产投资1375亿元，发挥了稳增长的决定性作用。其中，重点项目395个，完成投资850亿元，占总投资的62%；重点产业项目210个，占重点项目总数的53.1%；投资20亿元以上的重大项目32个，比2016年增加11个，项目结构明显优化，规模和质量都有较大提升。

（二）产业转型步伐加快。以供给侧结构性改革为主线，扎实推进“三去一降一补”，关闭煤矿23对、石膏矿山5家，粘土砖厂全部取缔，烟花爆竹生产企业全部退出。钢铁、水泥等产业“老树发新芽”，质量效益提升。高新技术产业增加值增长17%。六大高耗能行业占规模工业的比重，从41.5%下降到36.9%。万元GDP能耗下降5.3%。园区规模工业增加值占全市规模工业的比重，由62%提高到66.2%。三迅电子、电将军、上海星珂实业、三一中源等新技术新材料工业企业加快成长。现代金融、商贸物流、文化旅游等产业快速发展。湘中国际物流园、诚通物流园、五江·碧桂园等项目的主体功能区建成使用。全年接待游客3600万人次，旅游收入增长30%。互联网经济、总部经济、赛事经济、智能制造等新兴产业有新作为，陌陌、葛洲坝集团、中钰基金等在我市设立独立公司。农业现代化、产业化、机械化进程加快，新增农业合作社983个、市级以上龙头企业30家，农产品加工产值增长20%。新增规模以上企业280家，净增125家，其中高新技术企业总数达到85家，新增27家。三次产业结构调整为14：47.5：38.5。

（三）脱贫攻坚扎实有效。坚持“四出”“五进”“八不退”标准，按照“两个服气”要求，扎实开展精准识贫专项行动，排查出不符合贫困条件的错评对象21.02万人、符合条件的漏评对象11.14万人。制定了脱贫攻坚“四个意见”“十条禁令”，建立了“有事请找我”责任担当机制，在“三走访三签字”的基础上，设置了“三张联系卡”，狠抓产业、教育、金融、健康、劳务、水利、易地扶贫搬迁和贫困户危房改造等各项扶贫工作。全市减少贫困人口9.98万人，156个贫困村实现脱贫摘帽，在全国全省督查评比中取得优异成绩，涌现出了新化县油溪桥村、娄星区湘村万乐电商平台等先进典型。

（四）城乡环境明显改善。以“天蓝水净地绿三年行动计划”、湘江流域污染防治“一号工程”为总揽，全面推行河长制，推进孙水河保护立法，划定生态保护红线。抓好中央环保督察问题整改，交办的9个问题已解决7个，2个完成年度任务，245个信访问题全部提前办结。推进锡矿山地区环境综合治理，启动了砷固废资源化无害化工程技术中心建设。超额完成黑臭水体治理年度任务，全面完成450个村的环境综合整治，基本完成6个工业园区废水处理设施建设，淘汰黄标车7525辆。探索推进区域碳排放零增长创建活动，率先在全国地级市中完成碳普查和碳规划。中心城区空气质量优良率达到87.1%，地表水质、6个江河交界考核断面水质，以及3个“水十条”国家考核断面水质，达标率均为100%。智慧娄底建设加快推进，交通秩序、超限超载、农贸市场等专项整治成效明显。大力实施“综合交通提升三年行动计划”，益娄高速、娄新高速金竹山互通建成通车，完成干线公路改造106公里、农村公路提质改造680公里。全面启动高速公路沿线8个特色小镇试点建设，冷水江市禾青镇获批全国特色小镇。双峰县荷塘村等3个村获评第五届全国文明村镇。

（五）改革开放持续推进。9大类161项重点改革进展顺利。出台了加快金融产业发展、促进民间投资、补齐公共设施关键短板等政策文件。加快“放管服”改革，全面实施“多证合一”，行政审批事项精简70%，办理时限缩短50%。取消、降低或放开43项行政事业性和服务性收费。深入推进园区管理体制改革，市直1473项管理权限全部下放到娄底经开区。不动产登记制度、农村土地确权登记颁证等改革进展顺利。市本级公车改革全面完成。娄星区被征地农民“养老贷”、冷水江市社区警务网格化与社区矫正、涟源市刑释人员安置帮教、新化县先富村并贫困村共同致富、涟钢“三供一业”分离移交等改革，被国家和省里宣传推介，双峰县涉农资金整合改革被评为全省首届“管理创新奖”。坚持创新引领，高举开放大旗，突出招大引强，出台了坚持开放崛起战略建设开放强市的实施办法，制定了落实创新驱动发展战略建设科技强市的实施意见。成功举办第八届湘博会。全年内联引资605.3亿元，增长17.5%；实际利用外资4.69亿美元，增长17.8%；实现进出口总额80.4亿元，增长35.3%。

（六）民生事业保障有力。省定12个重点民生实事项目全面完成。全面小康实现程度达到83.4%。完成农村薄弱学校改造项目134个。娄星区获评全国义务教育发展基本均衡县（市、区）和全省教育强县（市、区）。全市53%的村完成文化服务中心建设，人均体育场地面积达到1.39平方米。完成城市棚户区改造26603户、农村危房改造12030户。租赁补贴、灵活就业人员缴存住房公积金等政策有效落实。智慧医疗有序推进，大病保险、大病救助全面落实，公立医院取消药品加成，建档立卡贫困户住院医疗费用医保报销比例提高10%。新增养老床位3216张。在全省率先对农村低保对象和社会兜底脱贫对象进行核查，农村低保标准提高至每人每月268元。

（七）社会大局总体稳定。加强安全生产源头治理，深入开展重点行业领域“十大战役”专项整治，生产经营性安全事故起数和死亡人数，分别下降60%和46%。坚持“政府牵头、司法主导、依法处置”思路，成立民间金融纠纷调解中心和中心城区打处非集中办案中心，稳妥化解民间融资风险。加强食品、药品、农产品安全监管，全年没有发生一般以上食品安全事件，全省民调群众满意度提高4.5个百分点。扎实开展“十大平安创建”活动，刑事、治安发案大幅下降，命案全部告破。全面开展了“无上访乡镇（街道）、无上访村（社区）”创建活动，圆满完成了1837个村、258个社区的换届选举工作。

一年来，我们认真学习贯彻党的十九大精神，深入推进“两学一做”学习教育暨责任心教育常态化制度化，出台“十条规定”，切实加强政府自身建设。制定了法治政府建设实施方案，充分发挥政府法律顾问作用，提高依法决策水平。坚持“零容忍”惩治腐败，深入开展“雁过拔毛”式腐败问题和“纠‘四风’治陋习”专项整治，立案1619件，给予党纪政纪处分1435人，组织处理55人。加强审计监督，经审计上缴财政2.16亿元。

主动接受人大及其常委会的法律监督和工作监督，自觉接受政协民主监督和社会舆论监督，充分尊重人大代表、政协委员建议意见。全年办结人大代表议案1件、建议189件，政协委员提案166件。所提问题得到解决或基本解决的145件，纳入计划正在解决的194件，一时难以解决做出解释说明的17件。老百姓特别关注的城区生活垃圾无害化处置正在按计划推进，学校大班额等问题正在有效化解。

狠抓民生实事落实落地，总体完成年初确定的10件民生实事。偏远山区中小学校WIFI无线网络全覆盖、长沙—娄底—新化低空旅游观光航线等2件，提前完成；城市棚户区改造、新增城镇就业和农村劳动力转移就业、打通中心城区“断头路”等3件，圆满完成；农村全科医生或助理全科医生驻村全覆盖、易地扶贫搬迁、湘江资江娄底流域截污和水体治理等3件，完成年度任务；中心城区城乡公交一体化项目列入了全国公交都市建设，购置了新能源纯电动汽车50辆，已开通2条线路；娄底大道娄双段主体工程基本完成，沿线慢行系统自行车道及中心城区国际马拉松赛道正在积极推进，娄底大道冷新段一标段（4.26公里）没有完成建设任务。对持续实施和未完工的项目，我们将作为今年的民生实事，继续予以推进。

国防动员和后备力量建设全面加强，军民融合深度发展，全民国防教育普及深化，驻娄解放军、武警、消防、公安和民兵预备役遂行任务能力持续提升。统计、人防、地震、气象、移民、供销社、公共资源交易、档案、机要、保密、地方志、对台、贸促、外事侨务、港澳、民族宗教、工商联、工会、共青团、妇女儿童、老龄、关工委、文联、科协、红十字会、慈善、残联、计生协、机关事务等各项工作，均取得了新的进步。

各位代表！

2017年，面对复杂多变的经济形势和多重压力叠加的严峻挑战，全市上下履职尽责，攻坚克难，创先争优，向省委、省政府和全市人民，交出了一份优异的“娄底答卷”，撸起袖子、扑下身子、干出样子的“娄底新作为”正在彰显，迎难而上、昂扬向上、蒸蒸日上的“娄底新气象”正在形成。这些成绩的取得，是市委总揽全局、坚强领导的结果，是市人大、市政协、娄底军分区有力监督、鼎力支持的结果，是各级各部门攻坚克难、团结奋进的结果，凝聚了全市人民和社会各界的智慧和力量，倾注了各级各部门广大干部群众的心血和汗水。在此，我谨代表市人民政府，向全市人民，向各民主党派、工商联、人民团体，向驻娄解放军和武警、消防官兵、政法干警，向中央、省驻娄单位，向所有关心支持我市发展的各界人士，表示衷心的感谢并致以崇高的敬意！

与此同时，我们也深刻感受到，发展中还面临不少的困难和问题：一是经济下行压力较大，去年大部分指标增速虽高于全省平均水平，但仍有少数指标没有完成预期目标。二是经济总量依然不大，县市区之间发展不平衡，园区产业集聚度、关联度不高，脱贫攻坚、安全生产、生态环保和民生事业等方面欠账较多。三是驾驭新经济的能力不够强，做规划、谋项目的水平不够高，实体经济、产业发展成效还不够明显，又好又大的产业项目不多，支撑经济发展、推进产业转型的后劲不足。四是思想观念落后，创新能力不足，开放程度不够，办事效率不高，社会诚信受损，“三强”问题突出，征地拆迁、控违拆违难，发展环境还不优。五是部分干部精神不振、作风不实，得过且过、消极应付情绪一定程度存在，贪污腐败、雁过拔毛等违规违纪行为时有发生，等等。对此，我们将牢牢坚持问题导向，不回避、不推诿、不畏难，坚持抓重点、补短板、强弱项，一个一个地加以解决。

二、2018年目标任务和工作重点

2018年，是贯彻落实党的十九大精神的开局之年，是改革开放40周年，是决胜全面建成小康社会、实施“十三五”规划承上启下的关键之年，也是我市推进七个“三年行动计划”的收官之年。党的十九大和中央、省委、市委经济工作会议，对今年和今后一段时期的经济社会发展，做出了科学判断和全面部署，我们一定要提高思想认识，端正发展理念，立足娄底实际，找准工作重点，科学谋划并全力抓好各项工作。

2018年政府工作的总体要求是：全面贯彻党的十九大精神，以习近平新时代中国特色社会主义思想为指导，加强党对经济工作的领导，科学把握“稳中求进”，坚持新发展理念，紧扣社会主要矛盾变化，按照高质量发展的根本要求，以供给侧结构性改革为主线，加速转型、奋力赶超，深入实施创新引领开放崛起战略，大力培育和发展实体经济，坚决守底线、奋力争上游，统筹推进稳增长、促改革、调结构、惠民生、防风险各项工作，在打好精准脱贫、污染防治、防范化解重大风险攻坚战方面取得扎实进展，推动经济社会持续健康发展。

2018年的主要预期目标是：地区生产总值增长8.5%左右，其中一、二、三产业分别增长3.5%、8%、11%左右；固定资产投资增长14.5%以上，规模工业增加值增长8.5%左右，社会消费品零售总额增长11.5%以上；一般公共预算收入增长8%以上；城乡居民人均可支配收入分别增长8.5%以上和11%左右；居民消费价格指数控制在3%以内；节能降耗完成省定目标任务。

围绕上述目标，重点抓好六个方面的工作：

（一）坚决打好“三大攻坚战”，决战全面小康

打好精准脱贫攻坚战。全力推进“脱贫攻坚三年行动计划”，严格按照“两不愁三保障”“三率一度”“三走访三签字”的工作要求，瞄准深度贫困村和特殊贫困人口，一手抓脱贫，一手抓巩固，切实提高脱贫质量。突出产业扶贫，坚持“四跟四走”基本路径，提升农业经营主体带动作用，扩大贫困群众的受惠面。采取民企联村、企事业单位结对帮扶和电商平台进农村等措施，有效解决农产品销路难题，提升农民持续增收能力。扎实推进金融、教育、医疗、住房、社会等扶贫工作，加强对五保人员、无劳动力家庭、因病因学返贫家庭的社会救助力度。全面完成易地扶贫搬迁任务。继续在村支书中开展争当“脱贫致富带头人”活动，在贫困户中开展“人穷志不穷，脱贫靠自身”自强自立行动，强力治惰、治懒、治脏、治赌、治愚，激发贫困群众内生动力。确保全年实现10万以上贫困人口脱贫、200个以上贫困村整体脱贫、双峰县脱贫摘帽。

打好污染防治攻坚战。深入推进“天蓝水净地绿三年行动计划”和“湘江保护治理第二个三年行动计划”，扎实开展全国第二次污染源普查，继续抓好中央环保督察反馈问题整改落实，加强大气、水、土壤污染防治，重点抓好锡矿山地区环境综合治理、涟钢周边环境综合治理、煤矿沉陷区整治等工作。以《孙水河保护条例》实施为契机，全面落实河长制，完成县级以上饮用水源地规范化整治和标准化建设，加强黑臭水体治理和关闭煤矿矿井废水治理，推进资水、涟水、孙水等流域综合整治。加快国家森林城市创建，统筹推进山水林田湖草生态保护和修复，完成造林绿化15万亩。坚持低碳绿色发展，积极推进杨市镇捕碳草种苗基地、锡矿山地区生态修复和异地碳捕集等项目，探索创建零碳区域。

打好防范化解重大风险攻坚战。加快社会诚信体系建设，依法严厉打击非法集资、高息揽储等违法违规金融活动，稳妥处置民间融资风险，坚决守住不发生区域性金融风险的底线。构建政府主导、市场运作、管理规范、风险可控的政府举债融资机制，坚决防范政府性债务风险。严格落实安全生产责任，坚决淘汰落后不安全产能，全力抓好重点行业领域安全整治，有效防控安全事故。全面落实“四个最严”要求，大力实施食品安全战略，开展省级食品安全城市示范区创建。加强基层社会治理，全面推进“四位一体”村级治理，继续推进“无上访乡镇（街道）、无上访村（社区）”创建。扎实推进“雪亮工程”建设，依法打击和惩治黄赌毒黑拐骗等违法犯罪活动，全面建设“平安娄底”。

（二）大力发展实体经济，推进产业转型

打造工业升级版。以全国产业转型升级示范区建设为总揽，深入实施“工业提振三年行动计划”，将建设“钢铁新城”作为转型升级的标志性工程来打造，深度对接“中国制造2025”和湖南“制造强省”战略，推动钢铁、水泥、有色等传统产业，农机、精细陶瓷等特色产业，新能源、新材料、高端装备制造、电子信息、生物医药等新兴产业，向集约化、高端化、智能化、绿色化方向发展。做好建链、延链、补链、强链文章，抓好钛合金、电动大皮卡等重大工业项目，壮大互联网经济、不锈钢、耐火材料、应急、农产品精深加工等产业，加快打造“1+5”千百亿产业集群，即板材深加工及汽车零部件千亿以上产业集群，新能源电池及储能产业、装配式建筑、精细陶瓷、军民融合、健康旅游等5个百亿以上产业集群。

发展现代服务业。全力推进“文化旅游及健康养老产业发展三年行动计划”，积极对接省“双百”工程，加快发展现代金融、商贸、物流、信息、健康、养老、家政等生产性、生活性服务业，在做大做强做实湘中国际物流园、诚通物流园、五江·碧桂园、利和宁邦城市广场等项目的基础上，着力打造一批名街、名店、名品、名商场。加快足球小镇、景观公路、马拉松赛道、慢行系统自行车道、自驾车房车营地等项目建设，支持紫鹊界梯田、曾国藩故居、湄江国家地质公园创建5A级景区。完善景区基础配套，大力发展赛事经济，推动全域旅游向纵深发展，确保全年旅游接待人次、旅游收入均增长18%以上。

推进农业现代化。坚持质量兴农、绿色兴农，坚守耕地保护红线，确保粮食总产量160万吨左右，蔬菜总产量150万吨以上。整合各类涉农资金，着力抓好农村水利、电网、宽带、安全饮水等基础设施建设。坚持和完善农村承包地“三权分置”，促进农村土地流转，加快农业机械化，力争农机综合化水平达到56%以上。积极培育新型农业经营主体，支持农业合作社公司化运作，确保新增2家以上销售过亿的龙头企业。全面开展“互联网+现代农业”“互联网+商贸流通”和“电子商务进农村”行动，构建现代农业产业体系、生产体系、经营体系。完善农业保险制度，增强农业抵御自然风险的能力。大力发展休闲、观光、体验型农业，促进三次产业融合发展，提高农业综合效益。

大力发展实体经济，必须以提高发展质量和效益为中心，大张旗鼓地鼓励大众创业、万众创新。进一步落实减税、降费政策，降低水、电、气、运等生产性成本。设立政府主导的产业发展基金和企业扶持基金，鼓励国有资本以多种方式入股民营企业，鼓励民间资本参与公共领域投资和重大项目建设，鼓励投融资平台参与产业发展。扎实推进“财源培植三年行动计划”，力争新增规模以上工业企业100家以上、服务业企业40家以上、农业企业50家以上。

（三）抓实项目建设，扩大有效投资

加强投资引导。全面开展“产业项目建设年”活动，以“项目建设大比拼”为抓手，积极对接国家165个重大专项和省“百千万投资促进计划”，大力实施“531”投资促进计划，确保工业投资、高新技术产业投资分别增长16%以上、20%左右，基础设施投资、生态环境投资、社会民生投资，分别增长15%、20%、18%左右。提高产业投资的比重和质量，确保产业投资占全部投资的比重提高5个百分点以上。

推进产业集聚。坚持政策向园区倾斜、要素向园区集聚、产业向园区集中，加快产城融合，加强园区标准厂房建设，切实提高园区承载能力。加快国家级高新区创建步伐，制定“一区多园”操作细则，支持“飞地经济”试点。确保园区工业增加值增长12%以上，上缴税收增长20%以上，园区工业占全市规模工业的比重达到68%以上。

坚持招大引强。全方位对接北上广、大长沙，紧盯世界500强、中国500强、民营500强企业，依托现有产业基础，开展差别化招商、产业链招商、小分队招商。办好第九届湘博会、第五届娄商大会，支持异地商会建设，满腔热忱招“娄商”、引“外商”，确保引进投资50亿元以上项目2个、30亿元以上项目5个、10亿元以上项目8个。实现内联引资增长14.5%以上，实际利用外资增长10%以上。

优化营商环境。规范市场秩序，重拳打击走私、制假售假、“三强”等行为。全面整治招商引资中的违约失信问题，切实维护投资者权益。建立金融机构参与项目谋划、建设、招商引资全过程机制，努力扩大信贷投放，确保融资规模增长15%以上。创新融资担保体系，鼓励企业上市融资、债券融资、股权融资，有效缓解中小企业融资难融资贵问题。落实市级领导挂点企业（项目）机制，切实提高招商引资项目签约率、开工率、竣工率、达产见效率。全面推行征地拆迁货币化安置，坚持先征拆再挂牌，切实解决因征地难、拆迁慢而影响项目落地的问题。深化“放管服”改革，强力推行“只跑一次”政务服务流程再造，进一步缩短涉企行政审批及服务事项办理时限，打造经济发展环境娄底“小气候”。

（四）加快改革创新，激发发展活力

加强改革攻坚。深化供给侧结构性改革，以处置“僵尸企业”为抓手，引导煤炭等过剩产能市场化退出，统筹做好职工安置、债务处置等工作。坚持“房子是用来住的，不是用来炒的”定位，推行多主体供应、多渠道保障、“租购并举”的住房制度改革。探索盘活用好闲置农房和宅基地办法，深化农村产权制度改革。加快万宝新区体制机制改革。认真抓好财税、金融、供销、国企、教育和卫生等领域改革。深化监察体制改革，圆满完成试点任务。

突出创新引领。实施财政科技经费倍增计划，创建共享实验室，重点支持3至5个重大科技项目。加快综合性服务平台、创新平台、工程技术研究中心、重点实验室建设，发挥国家精细电子陶瓷检测中心、中科创一芯磁实验室等创新平台的引领作用。推动企业与高校、科研院所加强战略合作，共建核心技术研发团队、科技创新平台。深入实施“湘中英才聚集”工程，想方设法引进人才、留住人才、用好人才。

推进开放崛起。实施外贸“破零倍增”工程，积极创建省级出口食品、农产品质量安全示范区，推动“娄企出海”“娄品出境”，确保外贸进出口总额增长10%以上。积极参与“一带一路”建设，对接“湘欧快线”，协助办好“一带一路”沿线国家农机博览会，参与国际产能合作。加快铁路口岸平台、保税仓库、海关特殊监管区、外贸综合服务机构建设，加强与海关、商检机构的合作，推进国际贸易“单一窗口”电子口岸建设，提高贸易投资自由化水平。

（五）统筹城乡建设，推动融合发展

建设品质中心城区。以创建全国文明城市为总揽，推进创文管卫工作制度化常态化。加快实施“中心城区提质扩容三年行动计划”，全面打通“断头路”，改造城中村，修建小游园，整治背街小巷。实施城市生态修复和城市修补工程，坚决打好控违拆违攻坚战。加快地下综合管廊建设，完成道路提质改造及风貌整治和路网完善扫尾工程。加快水府庙——孙水河风光带建设，开展城市增绿提质、增花添彩及亮化行动。完成一、二污水处理厂提质改造和三水厂建设。加强停车场、停车位建管，有效化解停车难题。加快“智慧娄底”建设，建成“云上娄底”大数据中心，推动城市标准化、精细化、网格化和智能化管理。

发展壮大县域经济。坚持规划先行，优化县市城区功能定位和产业布局，加强基础配套，提升承载能力。抓实园区经济、项目经济、产业经济，提升县域综合实力。支持冷水江市打造世界“锑都旗舰”、涟源市发展农产品深加工业和应急产业、新化县发展文化旅游业、双峰县做大做强农机产业，逐步形成“一县一品、各具特色、错位发展”的县域经济新格局，新增一批销售超100亿的主导产业和超50亿的特色产业集群。加快娄底通用、民用两个机场建设，推进龙琅、官新等高速公路建设，加快8个高速公路沿线特色小镇建设，形成县域经济新的增长点。

大力推进乡村振兴。按照产业兴旺、生态宜居、乡风文明、治理有效、生活富裕的总要求，以农业产业振兴为总揽，全面提高农村环境质量、农村治理能力和农民收入，加快建设“五美”乡村。建立职业农民制度，加强农村专业人才队伍建设，鼓励引导工商资本参与农业振兴，鼓励社会各界人士投身乡村建设。以文明村镇创建、省级美丽乡村示范创建为契机，规范农村建房，开展联村建绿。建好、管好、护好、运营好农村公路，抓好村级综合服务平台建设，推进“厕所革命”，加强村庄、中心镇、乡镇机关、居民院落等环境整治。加强农村公共文化建设，开展“勤俭持家”“勤劳致富”“移风易俗”和“艰苦创业”模范评选活动，培育文明乡风、良好家风、淳朴民风。通过不懈努力，让农业成为有奔头的产业，让农民成为有吸引力的职业，让农村成为安居乐业的美丽家园。

（六）坚持以人民为中心的发展思想，增进民生福祉

继续抓好民生实事。全面完成娄底大道冷新段建设，继续推进农村建制村全科医生或助理全科医生驻村全覆盖行动计划、湘江资江娄底流域截污和水体治理行动、易地扶贫搬迁等3件实事。今年新增6件：完成116条背街小巷第一期改造任务；全面推进农村垃圾分类处理；全面实施山塘清淤行动；全力化解“大班额”（中心城区新建4所、改扩建3所学校），中小学校基本消除66人以上超大班额；开工建设中心城区11个小游园；全面启动自然村通水泥（沥青）路建设工程，实现1900个左右自然村通水泥路。

大力发展社会事业。开展“技能培训+创业培训”，完善劳务对接机制，提高就业创业成功率。新增城镇就业3.6万人、农村劳动力转移3.5万人。推动城乡义务教育一体化发展，继续加强农村薄弱学校改造，加快公办幼儿园建设，推进娄底幼儿师范学校中升专，改善教学条件，提高教师待遇，让我们的孩子幼有所育、学有所教。深化医药卫生体制改革，加强基层医疗卫生体系建设，让人民群众方便看病、看得起病。积极应对人口老龄化，推进医养结合，新增养老床位1200张以上，加快建设老年大学，让老年人老有所依、老有所养、老有所乐。加快推进规划馆、公共图书馆、美术馆、体育学校、工会活动中心、基层综合文化服务中心等项目建设，完成农村广播“村村响”工程。

切实加强民生保障。全面实施全民参保计划，完善基本养老、基本医疗和失业、工伤、生育等保险制度。完成城市棚户区、农村危房改造省定任务。加快推进殡仪馆、救灾物资储备中心等项目建设。统筹城乡社会救助体系，完善最低生活保障制度。完善社会福利、慈善事业、优抚安置等制度，健全农村留守儿童和妇女、老年人关爱服务体系。发展残疾人事业，提升残疾康复服务水平。用心用情抓好公共厕所、公交站、垃圾堆放、街道井盖、群众文化广场等群众身边的小事，解决好老房子漏水、下水道堵塞、停水停电停气等群众家门口的实事，让群众有更多的获得感。

用好用活军民融合政策，大力发展国防科技产业。压减民兵规模，调整民兵结构，加强国防后备力量建设。做好退役军人安置和教育引导工作，将其打造成维护基层稳定的重要力量。扎实开展第四次全国经济普查，全面做好第八批全国重点文物保护单位申报，加快推进第二轮娄底市志修编。统筹抓好其它各项工作，力争每项工作创出新特色，迈上新台阶。

三、加强政府自身建设

打铁必须自身硬。完成全年目标，兑现庄严承诺，必须进一步加强自身建设，有效提高政府执行力和落实力。

（一）加强学习，增强本领。认真开展“不忘初心、牢记使命”主题教育，深入推进“两学一做”学习教育暨责任心教育常态化制度化，确保党的十九大精神在娄底落地见效。深入开展“大学习大调研大提升”活动，切实提高适应新时代、实现新目标、落实新部署的能力。旗帜鲜明讲政治，践行“七个带头”，不折不扣落实党中央国务院、省委省政府和市委的决策部署，做到雷厉风行、令行禁止。

（二）依法行政，科学决策。坚持办事依法、遇事找法、 解决问题用法、化解矛盾靠法，确保政府一切活动在法制的轨道上运行。全面落实“谁执法谁普法”责任，实现行政机关法律顾问、公职律师全覆盖。自觉接受人大及其常委会的法律监督和工作监督、政协的民主监督和社会舆论监督，认真听取人大代表、政协委员、民主党派、工商联、无党派人士、各人民团体的建议和意见，确保政府决策科学，体现人民意愿。

（三）求真务实，狠抓落实。对照中央指出的形式主义、官僚主义等“十种表现”，坚持问题导向，切实改进作风，以一身正气、一心为民、一专多能、一鼓作气、一抓到底、一往无前的责任与担当，推动责任落实、任务落实、项目落实、工作落实。

（四）清正廉洁，干净干事。认真贯彻中央八项规定精神，一以贯之纠“四风”、治陋习，严肃查处侵害群众利益的不正之风和腐败问题。加强行政监察和审计监督，着力营造“亲”“清”新型政商关系。落实容错纠错机制，治理“为官不为”，旗帜鲜明为公道正派、敢于担当、实绩突出的干部撑腰鼓劲。

各位代表！

决胜全面建成小康社会的号角已经吹响，让我们紧密团结在以习近平同志为核心的党中央周围，在省委、省政府和市委的坚强领导下，加速转型、奋力赶超，履职尽责、创先争优，为建设富饶美丽健康祥和的新娄底，为全面夺取新时代中国特色社会主义的伟大胜利而不懈奋斗！
